# An unusual variant of choledochal cyst: a case report

**DOI:** 10.1186/1752-1947-3-54

**Published:** 2009-02-09

**Authors:** Javaid Sadiq, Biplap Nandi, Kokila Lakhoo

**Affiliations:** 1 Oxford Children's Hospital, Oxford, UK

## Abstract

**Introduction:**

Choledochal cyst is an uncommon congenital disease of the biliary tract in the UK. There are five main types of choledochal cyst with several recognised sub-types. However, occasional variants do occur.

**Case presentation:**

We report a case of a female infant with an antenatally diagnosed choledochal cyst. The operative cholangiogram revealed an unusual intrahepatic biliary tree. The cyst was successfully excised and the infant is well at 18-months follow up.

**Conclusion:**

The anatomy should be clearly defined before surgical excision as abnormal variants can occur, which usually do not fit into the known classification types and subtypes.

## Introduction

Choledochal cysts are congenital abnormalities of the biliary system and consist of cystic dilatations of the extrahepatic biliary tree, intrahepatic biliary ducts or both. The incidence of choledochal cyst in western countries has been reported to be 1:100,000 to 1:150,000 [1]. However, it is 100 times more common in Japan with an estimated incidence of 1:1000 [2,3]. Choledochal cysts are classified into five main types with several subtypes. Anatomical configurations, which do not readily fall into the Todani modification, have been described. We report one such case in a 7-month female infant with antenatally diagnosed choledochal cyst, which does not fit into this classification system.

## Case presentation

**Figure 1:**
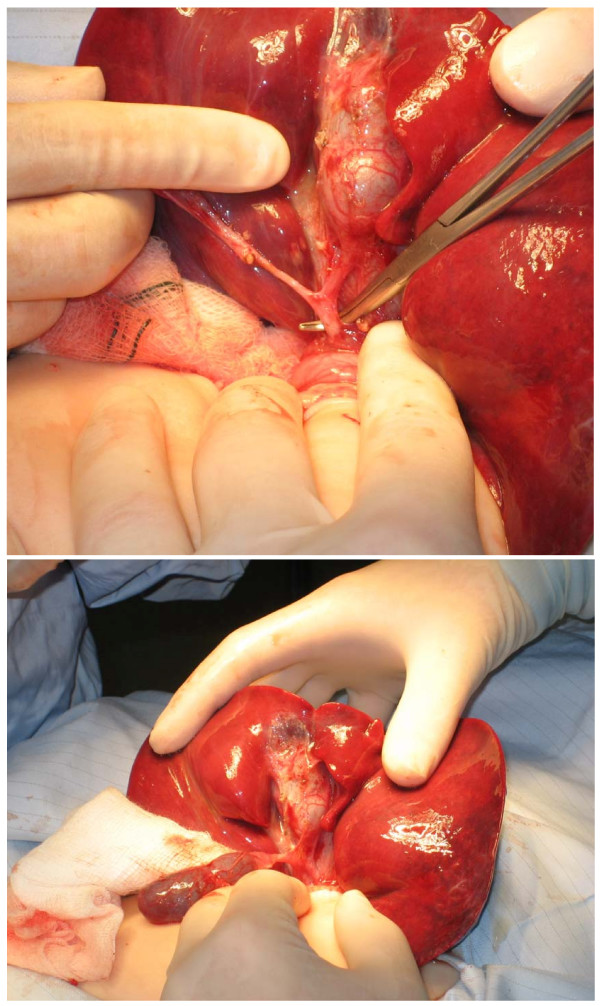
**Operative findings**.

**Figure 2:**
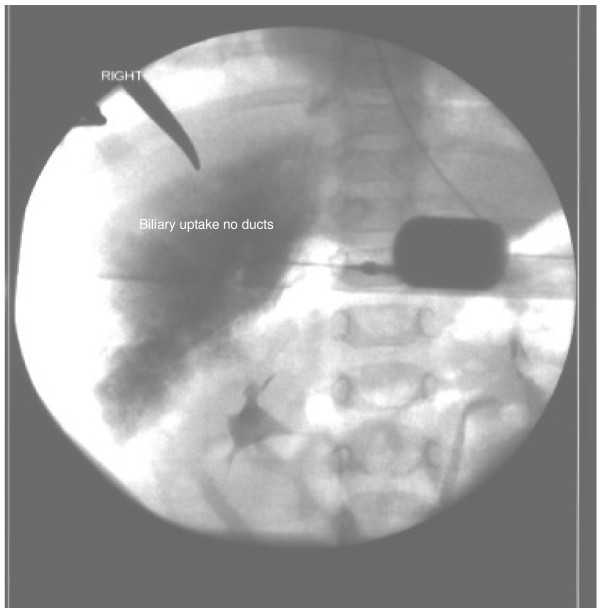
**Operative cholangiogram**.

A female Caucasian infant was born at term with an antenatal diagnosis of an abdominal cyst. Prenatal diagnosis could not confirm the exact origin of the cyst. A prenatal differential diagnosis of choledochal cyst, duplication cyst and omental cyst was given. Due to diagnostic difficulties, several post-natal scans were performed before the cyst was confirmed to be a choledochal cyst. The first two scans showed a cyst in the subhepatic area which was septated and loculated and was displacing the intrahepatic vessels. The common bile duct was found to be of normal calibre. There was no intrahepatic dilatation. It was reported to be a hepatic cyst or a choledochal cyst but with features not characteristic of either. She remained asymptomatic and once the diagnosis of a choledochal cyst was confirmed, an elective excision of the cyst was performed at the age of seven months. At laparotomy, a large 5 cm × 4 cm × 2 cm cyst was found at the level of the porta hepatis with normal hepatic and common bile duct (Figure 1) which did not fit with any of the current recognised classification types. An intra-operative cholangiogram demonstrated generalised biliary uptake in the liver with no evidence of intrahepatic biliary ducts (Figure 2). The cyst was completely excised and noted to be markedly inflamed and adherent to the hepatic capsule. A Roux-en-y porto-jejunostomy was performed and a liver biopsy taken. The patient made a good post-operative recovery. She later developed adhesive bowel obstruction and required a laparotomy for adhesiolysis. She went home on the 6th post-operative day after making a full recovery. At 3 monthly follow-up for the 1st year and subsequent 6 monthly follow-up, she has been well with normal liver and biliary functions and normal liver architecture on ultrasound scan.

Histopathology showed an inflamed cystic structure lined with stratified squamous epithelium and containing areas of probable metaplasia. The liver biopsy showed no evidence of biliary atresia.

## Discussion

Choledochal cyst is the cystic dilatation of extrahepatic bile ducts, with or without the dilatation of the intrahepatic biliary tree. It may be associated with biliary atresia [4].

Based upon the clinical and anatomic findings, in 1959, Alanso-Lej, Revor and Pessagno classified choledochal cysts into three main types [1]. This classification was further updated by Todani et al who described five main types with several subtypes [5-7]. Type 1 is the most common type present in 80% to 90% of cases. This involves the dilatation of the entire common hepatic or common bile duct or of segments of each. It is further subclassified into I A (cystic dilatation of the common bile duct), I B (focal segmental dilatation of the distal common bile duct) and I C (fusiform dilatation of both common hepatic and common bile duct). Type II is a diverticulum from the common bile duct. Type III is a choledochocele, which is found in the intraduodenal portion of the common bile duct. In type IV A, there are multiple dilatations of the intrahepatic or extrahepatic biliary tree but most commonly, a large solitary cyst of the extrahepatic duct is accompanied by multiple cysts of the intrahepatic ducts. In type IV B, there are multiple dilatations of the extrahepatic bile duct only. Type V is characterised by dilatation of the intrahepatic biliary radicles.

## Conclusion

Our patient is unusual in that the anatomy does not fit with any of the classical descriptions of choledochal cysts. The cholangiogram finding of a biliary blush was also unusual. The surgical management did not differ from a conventional choledochal cyst. However, surgeons should be aware of the possibility of variant anatomy in choledochal cysts, which is outside the normal classification for this condition.

## Consent

Written informed consent was obtained from the patient for publication of this case report and any accompanying images. A copy of the written consent is available for review by the Editor-in-Chief of this journal.

## Competing interests

The authors declare that they have no competing interests.

## Authors' contributions

KL was responsible for the study cocept & design & for final approval of the version for publication. BN was involved in patient care & helped in review of literature. JS was responsible for acquiring the data, review of literature and drafting & revising the manuscript.
